# Single nucleotide variants of receptor for advanced glycation end-products (*AGER*) gene: is it a new opening in the risk assessment of diabetic retinopathy?—a review

**DOI:** 10.1186/s43141-022-00297-5

**Published:** 2022-01-31

**Authors:** Pragya Ahuja, Abdul Waris, Sheelu Shafiq Siddiqui, Amit Mukherjee

**Affiliations:** 1 Institute of Ophthalmology, Jawaharlal Nehru Medical College and Hospital, Aligarh Muslim University, Aligarh, Uttar Pradesh India; 2 Rajiv Gandhi Centre for Diabetes and Endocrinology, Jawaharlal Nehru Medical College and Hospital, Aligarh Muslim University, Aligarh, Uttar Pradesh India

**Keywords:** Receptor for advanced glycation end products (*AGER*), single nucleotide variants (SNV), diabetic retinopathy, Gene marker, Risk factor

## Abstract

**Background:**

Diabetic retinopathy (DR) is a common microvascular complication of diabetes. There is strong evidence suggesting that DR has an inheritable component. The interaction between advanced glycation end products (AGEs) and their receptor is integral in the pathogenesis of diabetic retinopathy and its various complications, retinopathy being one of them.

**Overview and methodology:**

This review discusses the existing literature on the association between single nucleotide variants (SNV) of *AGER* gene and the risk of DR. It also discusses the current understanding of the AGE-*AGER* pathway in diabetic retinopathy. Through our article we have tried to consolidate all the available information about these SNVs associated with diabetic retinopathy in a succinct tabular form. Additionally, a current understanding of the AGE-*AGER* interaction and its deleterious effects on the cells of the retina has been discussed in detail to provide comprehensive information about the topic to the reader. A literature review was performed on PubMed, Cochrane Library, and Google Scholar for studies to find existing literature on the association between *AGER* gene SNVs and the risk, progression and severity of developing DR. This article will encourage scientific communication and discussion about possibly devising genetic markers for an important cause of blindness both in developed and developing countries, i.e., diabetic retinopathy.

**Result:**

Based on genetic studies done in Indian and Chinese population G82S(rs2070600) was positively associated with Diabetic Retinopathy. Patients of diabetic retinopathy in Caucasian population had −T374A(rs1800624) polymorphism. + 20T/A was found to be associated with the disease in a study done in UK. Association with G1704T(rs184003) was seen in Chinese and Malaysian population. A Chinese study found its association with CYB242T. -T429C(rs1800625) SNV was not associated with DR in any of the studies. G2245A(rs55640627) was positively associated with the disease process in Malaysian population. It was not associated in Malaysian and Chinese population. Promoter variant rs1051993 has also been found to a susceptible SNV in the Chinese population.

**Conclusion:**

While providing a comprehensive review of the existing information, we would like to emphasize on a large, multi-centric, trial with a much larger and varied population base to definitely determine these single nucleotide variants predisposing diabetic individuals.

## Introduction

Diabetes is a global epidemic that is projected to affect 642 million adults by 2040, with about 75% residing in low- and middle-income countries [1]. As per the 2019 report of the International Diabetic Federation, 351.7 million people of the working-age group (20 to 64 years) have been diagnosed or suspected to have diabetes in 2019 [2]. It is estimated that every third patient with diabetes has some form of diabetic retinopathy (DR) [3]. DR is emerging as a significant non-communicable disease leading to ocular morbidity. Its increased prevalence in the working-age group up to 50 years leads to poor quality of life and decreased psychosocial well-being [4]. So a careful understanding of the pathophysiology of this disease is imperative to help predict the future course of the disease and its associated complications.

Duration of diabetes is a significant risk factor for the development of DR. It develops in 2% of patients having type 2 diabetes for less than 5 years and in 25% of patients having the disease for 25 years or more [5]. However, once retinopathy develops in the patient, glycemic control becomes the most crucial factor in determining the course of the disease and helps control micro-vascular complications of type 2 diabetes mellitus [6].

Despite providing robust evidence for susceptibility to diabetic retinopathy, environmental risk factors do not account for complete risk susceptibility. In the DCCT trial, major environmental factors, glycosylated hemoglobin, and duration of diabetes explained only 11% of the variation in the risk of retinopathy [7]. In the WESDR study, a combination of three environmental factors, i.e., glycosylated hemoglobin, blood pressure and total cholesterol, could explain only a 10% variation in the risk of diabetic retinopathy [8]. Despite having reasonable glycemic control and a short duration of diabetes, some patients developed diabetic retinopathy later. While others with poor glycemic control and long duration of diabetes did not develop DR. [9, 10] All these factors thus raise the possibility of genetic factors determining the predisposition to diabetes and its severity. Previous studies demonstrating familial clustering of DR cases further reinforce the role of genetic factors in the pathogenesis of diabetic retinopathy [10]. DR is a complex multigenic interactive process, and any alteration in the genes related to various biochemical pathways is hypothesized to contribute to the development of DR.

AGEs are heterogeneous molecules formed by the non-enzymatic reaction of glucose-derived carbonyls with amino groups of amino acids. *AGER* binds to and internalizes low levels of AGEs for degradation in normal individuals. However, in people with diabetes, due to increased production and mutation in the *AGER* gene, there is an accumulation of AGEs. The interaction between AGEs and their receptor is integral in the pathogenesis of DR. When stimulated chronically, it triggers a downstream signalling cascade that causes the release of cytokines, chemokines, and growth factors-like VEGF. These act collectively and damage the cells of the retina. Through this article we aim to study single nucleotide variants (SNVs) and their association with the progression and severity of diabetic retinopathy. Any polymorphism in the gene for this receptor will alter this pathway and have an influence on the pathophysiology of diabetic retinopathy. Many researches have been done to determine these specific SNVs, which have further demonstrated a demographic and ethnic variation in their results. Through our article we have tried to consolidate all the available information about the SNVs associated with diabetic retinopathy in a succinct tabular form. Additionally, a current understanding of the AGE-*AGER* interaction and its deleterious effects on the cells of the retina has been discussed in detail to educate the reader about this topic as a whole. This article will encourage scientific communication and discussion about possibly devising genetic markers for this disease. To conclude, while providing a comprehensive review of the existing information, we would like to emphasize on a large, multi-centric, trial with a much larger and varied population base to definitely determine these single nucleotide variants predisposing diabetic individuals.

## Methods

### Identification of source

PubMed, Cochrane Library, Google Scholar, Embase, MEDLINE databases were searched extensively for the research studies in English, presenting outcomes on the study of various Single nucleotide variants (SNVs) of *AGER* gene and their association with the development and progression of DR.

### Method of evaluation

A critical assessment of the literature was performed. Preference was given to high-quality (high citation) papers, meta-analysis, longitudinal trials, and randomized control studies performed with correct statistical analysis and accurate methodology.

## Pathophysiology of diabetic retinopathy

### Role of oxidative stress

Oxidative stress induced by hyperglycemia plays a vital role in the pathogenesis of diabetic retinopathy. Altered expression of pro-inflammatory cytokines and inter-related pathways has been observed in retinal blood vessels. In the absence of an appropriate and effective endogenous antioxidant defence system, excessive reactive oxygen species (ROS) accumulation leads to activation of stress-sensitive intracellular signalling pathways, all of which promote cellular damage and diabetic complications [11]. Oxidative stress causes activation of various biochemical pathways, the polyol pathway, aldose reductase and protein kinase pathway. It also increases hexosaminidase pathway reflux and is associated with increased advanced glycation end product (AGE) formation [12, 13]. The conversion of glucose to sorbitol and fructose via the polyol pathway in chronic hyperglycemic conditions decreases intracellular antioxidant agents like NADPH and GSH [14].

**Fig. 1:**
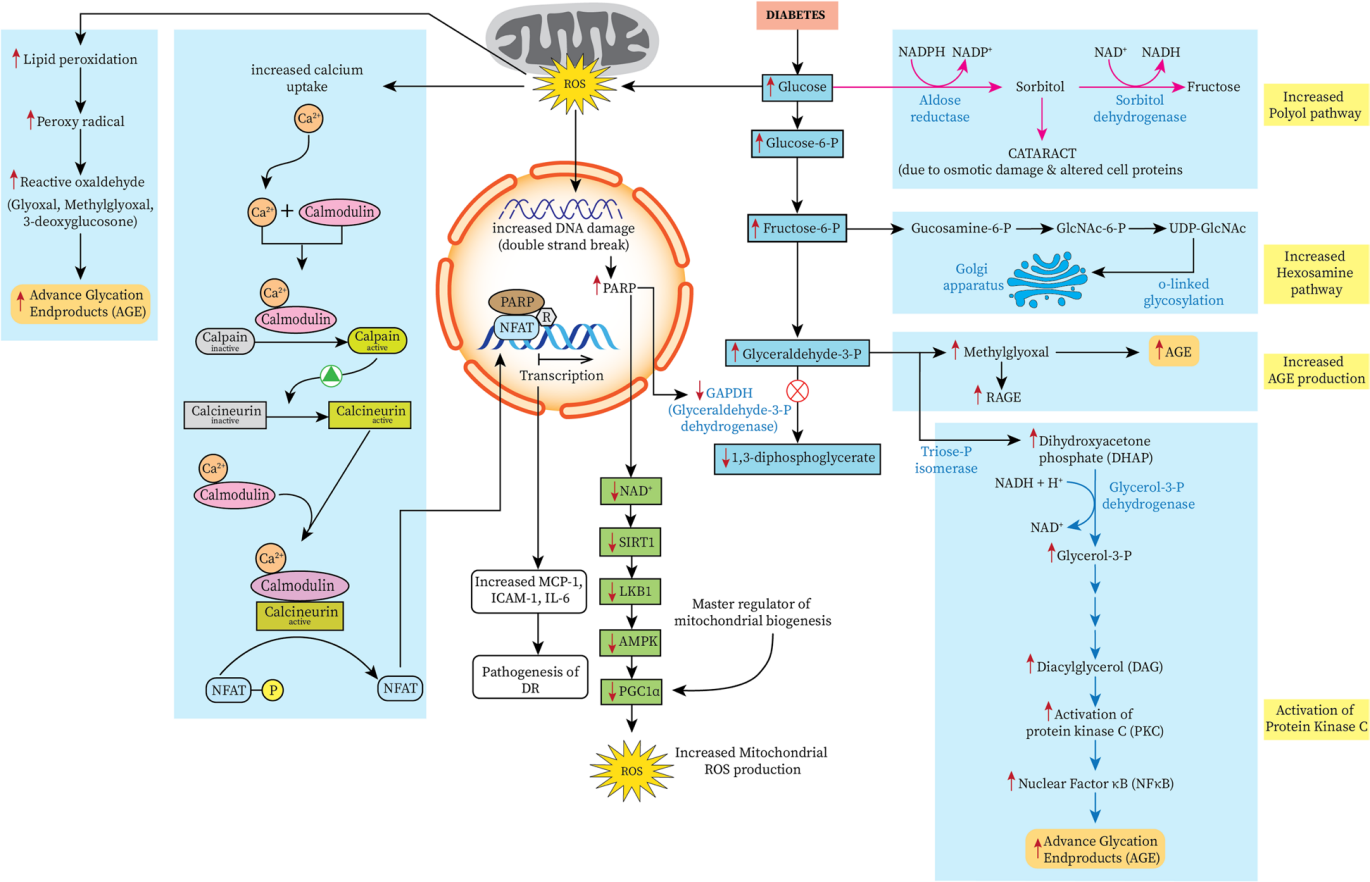
Hyperglycemia-mediated activation of various biochemical pathways. Reactive oxygen species generated due to hyperglycemia increase the transcription of various downstream signalling pathways by damaging the structure of DNA. This halts the normal pathway of glycolysis so that its substrates are diverted to other biochemical pathways. Glyceraldehyde 3 phosphate isomerizes to dihydroxyacetone phosphate (DHAP) that increases the production of advanced glycation end products. Activation of the hexosaminase pathway causes increased o glycosylation of glycolytic substrates. Activation of pyruvate kinase pathway, especially pyruvate kinase beta and NFAT pathway upregulation causes further activation of cytokines, chemokines, and interleukins, having deleterious effects on the cells of the retina, all of which is responsible for the changes of diabetic retinopathy. Activation of the polyol pathway leads to increased production of sorbitol and fructose, which are hyperosmotic agents and are implicated in the causation of diabetic cataract. As glyoxylase enzyme is generally overexpressed in the endothelium, AGE accumulation in the vessel wall is less. However, this detoxification system is deficient in people with diabetes, causing more pericyte apoptosis and thus accentuating DR pathology

Subsequent ROS accumulation inhibits glucose-6-phosphate dehydrogenase (G6PD) enzyme, which is necessary to reduce equivalents for the antioxidant system, thereby amplifying the oxidative stress. Further accumulation of NADH inhibits glyceraldehyde-3-phosphate, thereby increasing the precursors of the AGE pathway. This ultimately causes downstream activation of pyruvate kinase C. Chronic hyperglycemia not only activates these biochemical pathways, it also increases the levels of various cytokines, growth factors, endothelin-1 and angiotensin-2 of the RAAS system. The pyruvate kinase pathway is also activated, ultimately leading to hyperglycemia-induced cellular damage by inducing various chemical mediators. All these mechanisms lead to accelerated vascular damage associated with diabetes, leading to various macro- and micro-angiopathic complications like retinopathy, neuropathy and nephropathy [14]. Even though strict glycemic control can delay the effect and progression of diabetic retinopathy, it cannot stop its progression. This causes long-lasting damage to tissues even after glycemic control is achieved (Fig. 1).

### Advanced Glycation end products (AGEs)

**Fig. 2:**
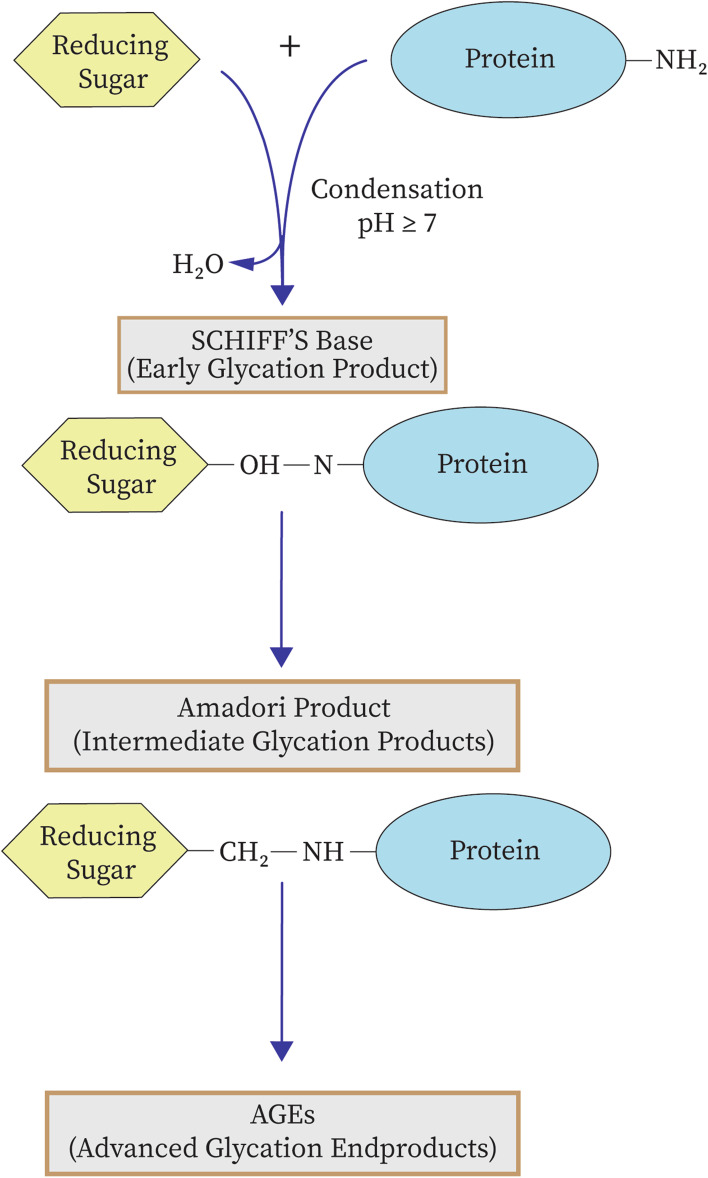
Maillard reaction for the formation of advanced glycation products

Advanced Glycation End products (AGEs) are heterogeneous molecules formed by the non-enzymatic reaction of glucose-derived carbonyls with amino groups of un-protonated lysine and arginine protein residues through a series of reactions forming Schiff bases and Amadori products [15]. This is a concentration dependant reaction in its early stages than later, thus explaining their increased formation in people with diabetes (Fig. 2).

AGEs and their intermediates usually accumulate in retinal blood vessels, glial cells, and neurons with age [15]. However, this deposition occurs at a faster rate in people with diabetes due to hyperglycemia, oxidative stress, and inflammation [16].

Over 20 different AGEs have been described to date, of which major groups are carboxymethyl lysine (CML), carboxyethyl lysine (CEL), pentosidine, glucosepane, methylglyoxal lysine dimer (MOLD), glyoxal lysine dimer, and glycolic acid lysine amide [15]. Hyperglycemia induced ROS overproduction is caused due to attachment of AGE to its receptor. Chronic hyperglycemia causes increased intracellular glucose accumulation causing glucose conversion to glyoxal while transforming the Amadori products to deoxyglucasone and producing methyl-glyoxal by fragmentation of glyceraldehyde 3-phosphate and dihydroxyacetone phosphate (DHAP) [17]. The majority of damage caused by AGEs is by glycolytic intermediates like glyoxal, methylglyoxal, and 3-deoxyglucose (3-DG). 3-Deoxyglucose is formed by non-oxidative rearrangement and hydrolysis of Amadori adducts and forms intermediates of the polyol pathway. In contrast, methylglyoxal is formed by the non-oxidative pathway of anaerobic glycolysis and oxidative decomposition of PUFA, fructose, ketone body catabolism, and amino acids like threonine [18, 19]. All these AGE intermediates then react with intracellular and extracellular proteins to form advanced glycation end products.

Not only diabetes, the role of AGEs has also been seen in male erectile dysfunction, pulmonary fibrosis, Alzheimer’s disease, reduced elasticity of the skin, and end-stage renal disease (ESRD), to name a few [20]. Histopathological studies have shown AGEs to accumulate in the kidney’s basement membrane causing glomerulosclerosis, coronary atheromas in atherosclerosis, amyloid-beta plaques of Alzheimer’s disease, and cartilage of rheumatoid arthritis [18].

AGEs like carboxyethyl-lysine (CEL), carboxymethyl-lysine (CML), and pentosidine have been localized in the retinal blood vessels, neurons, and glial cells of diabetic patients, and their levels were found to correlate with the degree of retinopathy [12, 21]. The accumulation of dicarbonyl, glycoxidation, or lipoxidation precursors forms either oxidative AGEs like CML and pentosidine or non-oxidative AGEs like DOLD (deoxy glucasone lysine dimer) from 3-deoxyglucose and MOLD (methyl glyoxal lysine dimer) from methylglyoxal. This phenomenon is called *carbonyl stress* and has been explained as one of the many pathological mechanisms responsible for accelerated vascular damage in diabetes [18].

Hyperglycemia induced ROS overproduction is mediated by AGEs interaction with its receptors like AGE-R1 (oligosaccaharyl transferase-48), 80K-H phosphoprotein (AGE-R2) *AGER*1/2/3, Galectin-3 (AGE-R3), CD36, and macrophage scavenger receptor (MSR-1 and 2) is implicated in the pathogenesis of diabetic retinopathy [14, 22].

## Receptor for advanced glycation end-products (AGER):

*AGER* is a multiligand member of the immunoglobulin superfamily [23]. Its truncated form, 35 kD transmembrane protein is expressed in many body cells like beta cells of the pancreas, lymphocytes, monocytes, neuronal cells, and endothelial cells in various tissues like the lung, liver, smooth muscle, endothelial cells, and brain [24, 25]. It acts as a scavenger and mediates intracellular signalling.

*AGER* has several distinct protein domains. The extracellular region where the following two domains form an integrated structural unit has a V type domain consisting of 96 amino acids and a C1 domain having 97 amino acids. The C2 domain having 90 amino acids, is a fully independent unit. It has a transmembrane domain with 20 amino acids that anchors *AGER* to plasma proteins, thereby helping in transducing signals to the cell interior. A highly charged short cytoplasmic domain having 43 amino acids has endogenous kinase activity and is responsible for intracellular signalling. A flexible linker protein connects the extracellular domain and C2 domain [26].

*AGER* exists in various functional variants, each of which has a tissue-specific regulation of expression. These are formed by alternative splicing or by the action of membrane-associated proteases. The *full-length AGER* (fl *AGER*) is also called *membrane-bound AGER* (m*AGER*). Its *N terminal truncated isoform* does not have a V domain, because of which it cannot activate the downstream signalling pathway. Instead, due to its ability to bind to m*AGER*, it may dampen its effect. This truncated form is also termed *tail deletion or Dominant-negative AGER* (Dn-*AGER*) [23, 27]. *Soluble AGER (sAGER)* is the C terminal truncated secretory isoform of receptor for advanced glycation end products. It is produced by alternate splicing of its encoding DNA at exon nine called *endogenous secretory AGER* (es*AGER*) or by the proteolytic cleavage of cell surface-bound m*AGER* [28, 29]. Since it lacks a transmembrane and cytosolic domain, it can be released into circulation easily. As *AGER* expression is increased by AGEs, its concentration is commonly used to indicate *AGER* gene expression in tissues. It scavenges *AGER* ligands and is responsible for the downstream signalling mediated by mediators like NFkB, SP-1, and AP-2 [26, 30].

The human gene for receptor of AGEs (*AGER*) is located on the short arm of chromosome 6, on locus 6p21.3 of MHC-III [31]. It has a 1.7-kb 5′ flanking region and 11 exons, 10 introns, and about 20 splice variants due to alternate splicing [23, 32]. Its 5′ flanking region regulates transcription. Apart from AGEs, *AGER* also binds to many different ligands like CML, S100s like S100A12, also called as (En-*AGER*), A-beta oligomers, HMGB1, LPA, CD11b/Mac1, C3a, which bind to V domain; A-Beta aggregates bind to the C1 domain and S100 A-Beta, which binds to the C2 domain in a dose-dependent manner [21, 27]. Not only is *AGER* production upregulated by these ligands, but also there is increased activation of the downstream signalling pathway mediated via various cytokines [33].

*AGER* expression is upregulated in many diseases like Alzheimer’s disease, diabetes mellitus, atherosclerosis, and rheumatoid arthritis [16, 34].

*AGER* binds to and internalizes low levels of AGE for degradation in normal individuals, thus protecting us from its deleterious effects. However, in people with diabetes, due to increased production and mutation in the gene for receptor of advanced glycation end products, there is an accumulation of AGE and receptor-mediated activation and secretion of various cytokines. These cytokines activate various signal transduction pathways like protein kinase C, JAK-STAT tyrosine phosphorylation, PI3′K recruitment to RAS, which creates a positive feedback loop responsible for long term side effects of hyperglycemia via increased NFkB and AP-1 transcription [32, 35]. Levels of glyoxylase enzyme (GLO1), responsible for AGE detoxification, are reduced in aged and diabetic tissues.

AGEs cause damage to target cells and cause a plethora of complications by many mechanisms:

### AGE-AGER interaction on extracellular matrix (ECM)

**Fig. 3:**
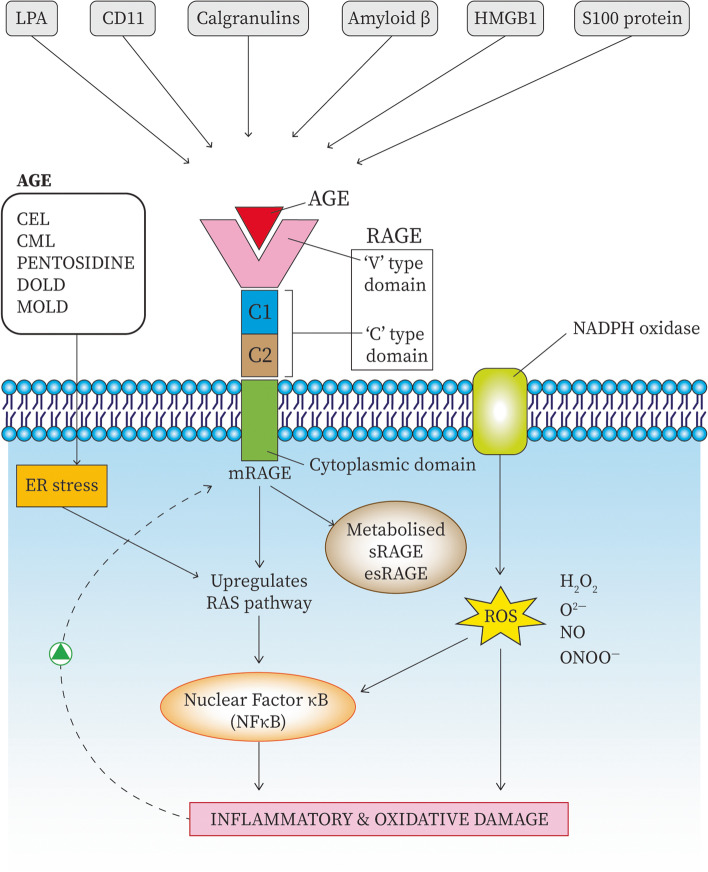
Receptor for AGE interacts not only with AGEs but also with other ligands like LPA, CD11, calgranulins, amyloid beta, HGMB1, and S100 protein. The interaction of AGEs with AGER initiates a downstream signalling cascade. The primary mediator for the inflammatory pathway is the ultimate induction of NFkB, which causes oxidative and inflammatory damage. Hyperglycemia associated oxidative ER stress and reactive oxygen species production induce NFkB. This inflammatory and oxidative damage thus caused creates a positive feedback loop causing further AGER activation

As advanced glycation is a slow process, it generally affects long-lived proteins like those of the extracellular matrix (ECM) and basement membrane. Altered AGE modification of sub-endothelial ECM components like collagen and laminin alters their interaction with other matrix proteins, and integrin receptors like integrin α2β1 [36] such that they do not respond to biological signals anymore. AGE acts on the intercellular junctions between various extracellular molecules formed by occludin, claudin and zonula occludens family and adhering junctions mediated by VE-Cadherin, affecting the junctional complexes stability. This affects the integrity of the blood-retinal barrier and makes the vessels leaky [37]. Sometimes, altered extracellular matrix components due to covalent cross-linking traps the circulatory proteins, affecting cellular traffic and cause narrowing of vessel lumen leading to vasculopathy [18, 38]. All these mechanisms get amplified due to increased AGE-*AGER* interaction, such that normal effector cells cannot perform reparative functions (Fig. 3).

### AGE-AGER mediated release of various cytokines and ligands

AGEs decrease the elasticity of blood vessels and deplete Nitric Oxide (NO), which has endothelium-dependent vasodilatory and anti-proliferative effects on the vascular smooth muscle [18]. Increased expression of intracellular *AGER*s and its major endogenous ligands is seen, which bind to each other and induce receptor-mediated production of deleterious gene products like ROS, NF-kB, and AP1 [24]. It also leads to increased production of cytokines like TNF-α, Interleukin-1β, and Interleukin-1α, thus responsible for hyperpermeability and growth factors like vascular endothelial growth factor (VEGF) [14, 27, 39]. Interleukin recruitment causes microglial activation and monocyte infiltration in the diabetic retina [40]. It has been hypothesized that AGE-*AGER* interaction causes VEGF over-expression by NADPH oxidase mediated ROS production, which further causes activation of redox-sensitive transcription factor NF-kB via the RAS-MAP kinase pathway [15, 40]. NF-kB modulates various genes like IL-6, TNF-α, ICAM-1, VCAM-I, and monocyte chemotactic protein–I (MCP-I) [22, 24]. Upregulation of VEGF causes endothelial mitogenesis, neovascularization and vascular leakage due to increased endothelial permeability [41].

### AGE-AGER interaction has a pro-coagulant effect

AGEs on attaching to the cell contribute to increased vascular permeability, pro-coagulant activity, and adhesion molecule expression. There is reduced platelet survival, increased platelet aggregation and altered levels of coagulation factors like anti-thrombin 3, tissue factor, thrombomodulin, and fibrinolytic inhibitor plasminogen-activator inhibitor-1(PAI-1) [42]. Various intracellular adhesion molecules, as mentioned above get activated due to AGEs and cause adhesion and transmigration of leucocytes to the endothelium. This leads to oxidative stress due to further polymorphonuclear leucocyte (PMNL) recruitment. When this cycle happens multiple times, there is endothelial loss and more activation of platelets and fibrin at that time [37]. These patients have alterations in von williebrand factor (vWf) , platelet function, and coagulation factors like factor VII [25]. All these contribute to the development of a pro-coagulant hypoxic state, thus contributing to the development and progression of vascular complications [24].

### AGE-AGER interaction’s effect on oxidative stress

On the one hand, AGEs increase reactive oxygen species (ROS) formation, while ROS also induces formation of AGEs. Their receptor binding causes tyrosine phosphorylation of JAK-STAT signal transducers, recruitment of phosphatidyl inositol 3′ kinase (PI3K) to RAS, and protein kinase C (PK-C) activation. Under normal conditions, *AGER* upregulation inhibits the activity of NADPH oxidase which decreases the formation of ROS. However due to persistent, long term exposure to AGEs *AGER* expression is altered because of which there is an increase in inflammation and oxidative stress [19].

### Pathogenesis of AGE-AGER interaction

AGEs are toxic to retinal capillaries [43]. They induce retinal pericyte apoptosis, osteoblastic differentiation, and calcification [37, 44, 45]. Vascular leakage is seen in these patients due to its increased endothelial permeability, pericyte loss, and pro-coagulant effect [22].

Muller cells contribute to the regulation of vital features of early retinal vasculature like homeostasis and vascular permeability. Due to the increased expression of *AGER* in these cells in people with diabetes compared to non-diabetics, muller cells are hypothesized to act as a regulator of diabetic retinopathy [46].

Microvascular changes like vessel wall thickening and pro-coagulant effect cause vaso-occlusion and induction of growth factors like VEGF, which leads to angiogenesis and neo-vascularization. The release of angiogenic growth factors like VEGF destroys the blood–retinal barrier causes retinal edema, neovascularization, hemorrhages, micro-aneurysms, and vessel occlusion. Recruitment of monocytes to sites of AGE accumulation brings about further endothelial dysfunction [37].

*AGER*-mediated cellular stimulation and ligand receptor activation leads to creation of a positive feedback loop thereby causing an increased expression of the receptor and thereby amplifying its downstream *AGER*-mediated signalling cascade. Thus, to put a stop to this cycle, mechanisms need to be devised to stop ligand binding to the receptor [27].

**Fig. 4:**
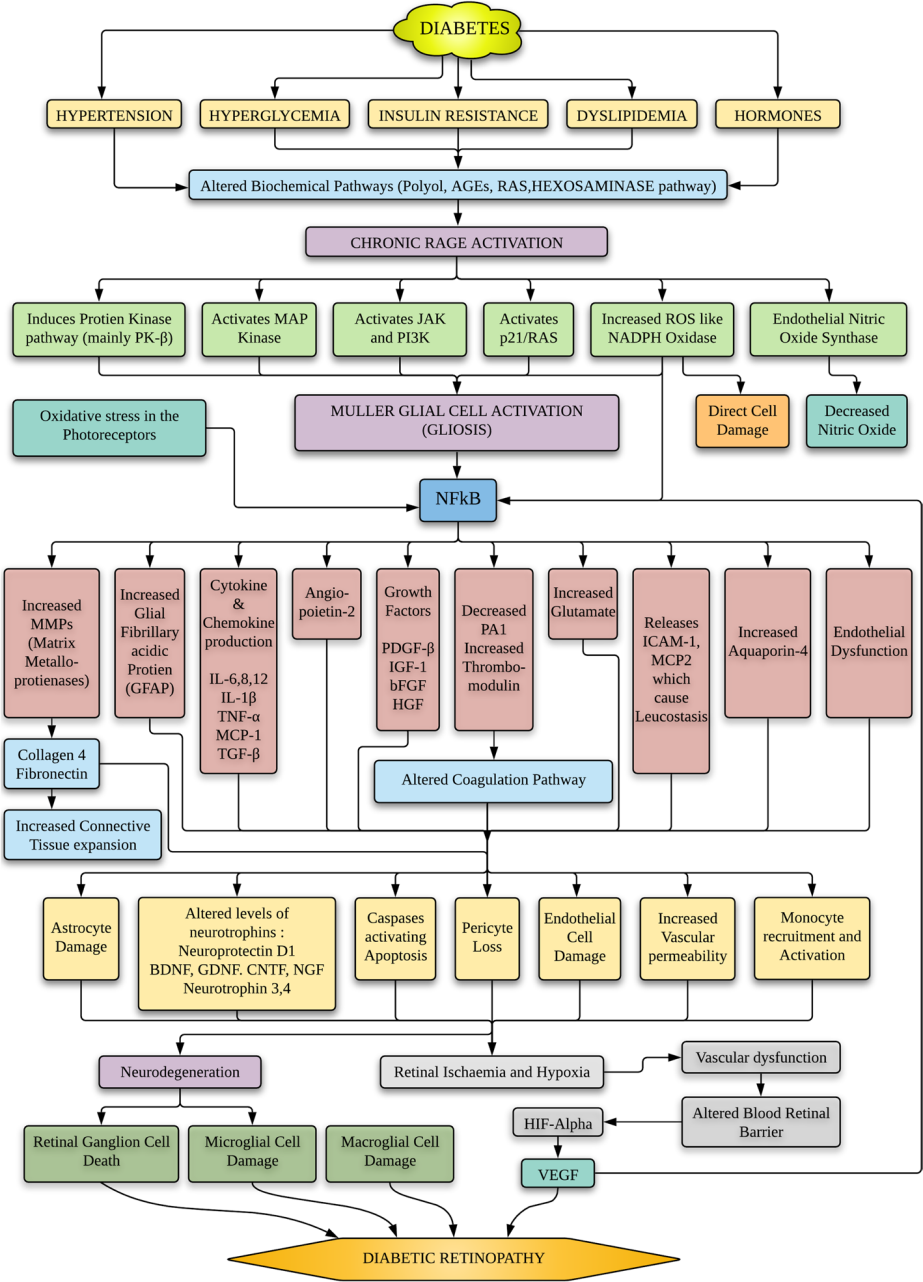
Downstream cascade of various chemokines, cytokines, and inflammatory mediators that causes damage to various retinal cells causing diabetic retinopathy

Inherited differences, mutations, and polymorphisms in key regions of the *AGER* gene may play an important role in AGE-*AGER* binding and altering their downstream signalling pathway, thus altering the future course of this disease [16, 35] (Fig. 4).

Over 30 polymorphisms are known in the *AGER* gene, most of which are Single nucleotide variants (SNVs), leading to altered function and expression of *AGER* if present in its regulatory region. It is essential to understand which of these gene variants is associated with the development of retinopathy as knowledge of these might help in its early detection in patients with specific SNVs, devise rapid diagnostic kits, and formulate therapeutic plans in the future. Many studies have been done to correlate the association between DR with genetic polymorphism in the *AGER* gene, but all these studies have had inconsistent results. A systematic assembly of the various *AGER* gene polymorphisms that have been studied and their variation with ethnicity will help us localize which polymorphisms are associated with the pathogenesis of diabetic retinopathy Table 1.

**Table 1:** Various SNVs in *AGER* gene and their association with diabetic retinopathy

S. No.	Researchers	Year	Subjects	Region	Genotype studied	Conclusion
1	Limei Liu, KunsanXiang et al. [47]	1999	156 type 2 diabetics	China	G82S (rs2070600) “NM_001136.5:c.244G>A NP_001127.1:p.Gly82Ser”	Not associated
2	Barry I. Hudson, Max H. Stickland et al. [42]	2001	215 [106 with DR 109 without DR]	Leeds, UK	1. -T429C (rs1800625) “NG_029868.1:g.4658T>C NC_000006.11:g.32152442A>G NT_167244.1:g.3467183A>G” 2. -407 to -345 deletion 3. -T374A(rs1800624) “NT_167249.2:g.3500817T>G NC_000006.12:g.32184610A>G” 4. +20T/A	Not associated Associated Associated Associated
3.	Govindasamy Kumaramanickavel, Vedam Lakshmi Ramprasad et al. [20]	2002	200 [100 with retinopathy 100 without retinopathy]	South Indian population at Sankara Netralaya Hospital , Chennai	G82S(rs2070600) “NM_001136.5:c.244G>A NP_001127.1:p.Gly82Ser”	Positively associated – low risk allele
4	XuJiXiong, XuBilin et al. [48]	2003	569 subjects (212 non diabetics 357 type 2 diabetics–205 with DR and 152 without DR)	China	1. -T429C (rs1800625) “NG_029868.1:g.4658T>C NC_000006.11:g.32152442A>G NT_167244.1:g.3467183A>G” 2. - T374A (rs1800624) NT_167249.2:g.3500817T>G NC_000006.12:g.32184610A>G	Not associated Not associated
5	Keiji Yoshioka, Toshihide Yoshida et al. [49]	2005	268 diabetics 98 controls	Japan	1. G1704T (rs184003) “NG_029868.1:g.6804G>T NM_001136.4:c.822+49G>T” 2. G82S (rs2070600) NM_001136.5:c.244G>A NP_001127.1:p.Gly82Ser	Not associated Not associated
6	S Ramprasad V Radha et al. [24]	2006	149 normal glucose tolerant subjects and 379 type 2 diabetics (189 DM without DR and 190 DM with DR)	South India	1. -T429C (rs1800625) “NG_029868.1:g.4658T>C NC_000006.11:g.32152442A>G NT_167244.1:g.3467183A>G” 2. -T374A ( rs1800624) “NT_167249.2:g.3500817T>G NC_000006.12:g.32184610A>G”	Not Associated Modest Association in NPDR group
7	B Fan, D.Y.Wang et al. [50]	2006	554 diabetics [120: With PDR 208: With NPDR] 226: Without DR	Hong Kong, China	G1704T(rs184003) polymorphism coinheritance with CYBA-C242T NG_029868.1:g.6804G>T NM_001136.4:c.822+49G>T	Associated
8	Suganthalakshmi Balasubbu, Periasamy Sundaresan et al. [38]	2010	701 (345 with DR 356 without DR)	South Indian population at Aravind Eye Hospital, Madurai, Tamil Nadu, India, and National Eye Institute, NIH, Bethesda, MD.	1. G82S(rs2070600) “NM_001136.5:c.244G>A NP_001127.1:p.Gly82Ser” 2. -T374A (rs1800624) “NT_167249.2:g.3500817T>G NC_000006.12:g.32184610A>G” 3. -T429C(rs1800625) “NG_029868.1:g.4658T>C NC_000006.11:g.32152442A>G NT_167244.1:g.3467183A>G	Associated Not Associated Not Associated
9	Zhi Xiang Ng, Umah Rani Kuppuswamy et al. [51]	2011	342 type 2 diabetics (171 with retinopathy 171 without retinopathy 235 unrelated healthy controls)	Malaysia	1. -T429C(rs1800625) “NG_029868.1:g.4658T>C NC_000006.11:g.32152442A>G NT_167244.1:g.3467183A>G” 2. -T374A (rs1800624) “NT_167249.2:g.3500817T>G NC_000006.12:g.32184610A>G”	Not associated Not associated
10	Zhi Xiang Ng, Umah Rani Kuppuswamy et al. [40]	2011	342 type 2 diabetics (171 with retinopathy 171 without retinopathy) 235 unrelated healthy controls	Malaysia	G2245A (rs55640627) NG_029868.1:g.7344G>A NM_001136.4:c.964+268G>A	Associated
11	Peipei Kang, Changwei Tian et al. [51]	2012	Meta-analysis of 29 articles	–	1. G82S(rs2070600), NM_001136.5:c.244G>A NP_001127.1:p.Gly82Ser 2. G1704T (rs184003), NG_029868.1:g.6804G>T NM_001136.4:c.822+49G>T 3. -T429C(rs1800625) NG_029868.1:g.4658T>C NC_000006.11:g.32152442A>G NT_167244.1:g.3467183A>G	Not Associated Not associated Not associated
12	Dongqing Yuan, Donglan Yuan et al. [52]	2012	Meta-analysis	–	1. -T374A (rs1800624) NT_167249.2:g.3500817T>G NC_000006.12:g.32184610A>G 2. G82S(rs2070600) NM_001136.5:c.244G>A NP_001127.1:p.Gly82Ser 3. -T429C(rs1800625)	Protective Associated Not Associated
13	Z.X.Ng, U.R.Kuppusamy et al. [47]	2012	283 (98 with retinopathy and 185 unrelated healthy subjects)	Malaysia	1. G82S(rs2070600) NM_001136.5:c.244G>A NP_001127.1:p.Gly82Ser 2. G1704T (rs184003) NG_029868.1:g.6804G>T NM_001136.4:c.822+49G>T 3. A2184G (rs3134940)	Not associated Not associated Not associated
14.	Li Yang, Qunhong Wu et al. [53]	2013	1040 type 2 DM patients 372: With DR 668: Without DR	China	G82S (rs2070600) NM_001136.5:c.244G>A NP_001127.1:p.Gly82Ser SS genotype was a risk factor for DR.	Associated
15	Weihong Yu, Jingyun Yang et al. [54]	2016	Meta-analysis	–	1. G82S(rs2070600) “NM_001136.5:c.244G>A NP_001127.1:p.Gly82Ser” 2. -T374A (rs1800624) “NT_167249.2:g.3500817T>G NC_000006.12:g.32184610A>G”	Associated Associated
16	Jian Li, Wei Cai et al. [22]	2016	943 type 2 diabetics 285: Patients with DR 658: Patients without DR	China	1. A2184G (rs3134940) “NG_029868.1:g.7284A>G NM_001136.4:c.964+208A>G NC_000006.11:g.32149816T>C NT_167244.1:g.3464557T>C”	Not associated
17	Dan Tao, Xuancheng Mai et al. [55]	2017	Meta-analysis	China	2. -T374A (rs1800624) “NT_167249.2:g.3500817T>G NC_000006.12:g.32184610A>G”	Associated
18	Wen-Ying Fan, Hong Gu et al. [31]	2020	1461 diabetics 567 selected for genotyping Diabetics with PDR: 97 Diabetics with NPDR: 217 Diabetics without retinopathy: 255	China	Promoter variant rs1051993 “NG_029868.1:g.3666G>T NC_000006.11:g.32153434C>A NT_167244.1:g.3468172C>A”	Associated

### Unanswered questions and future needs

Although there is strong evidence to suggest that DR is a heritable trait none of these polymorphisms have however received widespread public acceptance due to the polygenic and multifactorial nature of DR, in addition to the influence of various environmental factors. Insufficient sample sizes with inadequate phenotypic characterization of subjects to explain diabetic retinopathy is the reason for limited success in this field. Further prospective studies with some standardizations of phenotypic variables are necessary to confirm the association between SNV in the RAAGE gene and pinpoint which specific SNVs are associated with an individual’s predisposition to diabetic retinopathy and the progression of its severity. It will also help in further understanding the influence of ethnic background on this association. This polymorphism study may also help us understand the precise pathogenic mechanisms involved with DR. Not only will this specific polymorphism help diagnose such high-risk patients, but it will also help in devising specific gene therapies for such high-risk subjects in the coming future. This might help halt the onset and progression of the disease and could be a path-breaking development in our attempt to save the world from blindness due to DR.

## Conclusion

Receptor of advanced glycation end products gene on chronic stimulation by hyperglycemia acts at transcriptional level, to release various proinflammatory interleukins, chemokines and growth factors. This damages the glial cells in the retina and cause pericyte apoptosis and extensive endothelial vascular damage.

Based on genetic studies done in Indian and Chinese population G82S(rs2070600) was positively associated with Diabetic Retinopathy. Patients of diabetic retinopathy in Caucasian population had −T374A (rs1800624) polymorphism. +20T/A was found to be associated with the disease in a study done in the UK. Association with G1704T(rs184003) was seen in Chinese and Malaysian population. A Chinese study found its association with CYB242T. -T429C(rs1800625) SNV was not associated with DR in any of the studies. G2245A(rs55640627) was positively associated with the disease process in Malaysian population. It was not associated in Malaysian and Chinese population. Promoter variant rs1051993 has also been found to a susceptible SNV in the Chinese population.

Despite the comprehensive work and analysis done on finding the association between *AGER* gene SNV and DR risk, none of the identified polymorphisms have achieved widespread acceptance as a marker of high risk of diabetic retinopathy. These studies still have limitations and should be interpreted cautiously. The discrepant findings might be due to the differences in the statistical power, demographic variations, type of diabetic retinopathy, and the genetic and environmental background. Diabetic retinopathy is a multigenetic and multifactorial disease. Most of the studies included here were cross sectional studies, so the influence of *AGER* gene SNVs on the progression of DR was difficult to assess. Our *AGER* gene SNV analysis not only provides a deeper understanding of diabetic retinopathy pathogenesis, but also implies novel targets for DR gene therapy. The application of pharmacogenetic principles appears to be a promising strategy in attenuating the diabetes mellitus-mediated retinal vascular complications. Genetic marker screening will help identify the people at risk, slowing the disease progression and help reduce the micro-vascular complications, disability, and mortality rates.

Given the increase in DR incidence and presence of multiple research centers, uniform guidelines need to be devised for data collection and genetic analysis as a part of the larger collaboration between different countries representing varied ethnic groups. It is hoped that over the next few years, promising therapies need to be evaluated in clinical context, thereby reducing the medical and economic burden of DR and its associated blindness.

## Data Availability

Not applicable

## Abbreviations

1,3–BPG: 1,3-Biphosphoglycerate
ADP: Adenosine diphosphate
AMP: Adenosine mono phosphate
DAG: Diacyl glycerol
DHAP: Di-hydroxy acetone phosphate
DNA: Deoxyribonucleic acid
DR: Diabetic retinopathy
ECM: Extra-cellular membrane
eNOS: Endothelial nitric oxide synthase
ERK-1/2: Extracellular signalling regulated protein kinase
GADPH: Glyceraldehyde-3 phosphate dehydrogenase enzyme
GFAT: Glutamine fructose-6-phosphate transaminase
Glc-6-P: Glucosamine-6-phosphate
GlcNAc-6-P: N-Acetylglucosamine-6-phosphate
HIF-alpha: Hypoxia inducible factor-1-alpha
ICAM-1: Intracellular cell adhesion molecule
IGF: Insulin-like growth factor
IL-1,6: Interleukins
iNAD: Intracellular nicotinamide adenine dinucleotide
JAK: Janus kinase
LKB-1: Liver kinase B1
MAP kinase: Mitogen-activated protein kinase
NFAT: Nucleic factor of activated T cells
NADP^+^: Nicotinamide adenine dinucleotide phosphate
NADPH: Reduced nicotinamide adenine dinucleotide phosphate
NADPH oxidase: Nicotinamide adenine dinucleotide phosphate oxidase
NFkB: Nuclear factor kappa B
NO: Nitric oxide
0-Glc: O-linked glucosamine
p21RAS: RAS p21 protein activator-1
PA-I: Plasminogen activator inhibitor-1
PARP-1: Poly ADP ribose polymerase-1
PDGF-β: Platelet-derived growth factor- beta
PI3K: Phosphoinositide-3-kinase
PK-β: Pyruvate kinase-beta
PPAR-alpha-1: Peroxisome proliferator-activated receptor gamma cofactor-1-alpha
*AGER*: Receptor for advanced glycation end products
ROS: Reactive oxygen species
SIRT-1: Sirtuin-1
SNV: Single nucleotide variants
TCA: Tri-caboxylic citric acid cycle
TGF- β: Transforming growth factor beta
TLR4: Toll-like receptor 4
TNF-alpha: Tumor necrosis factor-alpha
UDP-GlcNAc: Uridine diphosphate N-acetyl glucosamine
VCAM-1: Vascular cell adhesion protien-1
VEGF: Vascular endothelial growth factor
